# Magnetic Resonance Spectroscopy in the Diagnosis of Dementia with Lewy Bodies

**DOI:** 10.1155/2014/809503

**Published:** 2014-07-06

**Authors:** Radoslaw Magierski, Tomasz Sobow

**Affiliations:** ^1^Department of Old Age Psychiatry and Psychotic Disorders, Medical University of Lodz, Czechoslowacka 8/10, 92-216 Lodz, Poland; ^2^Department of Medical Psychology, Medical University of Lodz, Sterlinga 5, 91-425 Lodz, Poland

## Abstract

Dementia with Lewy bodies (DLB) is considered to be the second most frequent primary degenerative dementing illness after Alzheimer's disease (AD). DLB, together with Parkinson's disease (PD), Parkinson's disease with dementia (PDD) belong to *α*-synucleinopathies—a group of neurodegenerative diseases associated with pathological accumulation of the *α*-synuclein protein. Dementia due to PD and DLB shares clinical symptoms and neuropsychological profiles. Moreover, the core features and additional clinical signs and symptoms for these two very similar diseases are largely the same. Neuroimaging seems to be a promising method in differential diagnosis of dementia studies. The development of imaging methods or other objective measures to supplement clinical criteria for DLB is needed and a method which would accurately facilitate diagnosis of DLB prior to death is still being searched. Proton magnetic resonance spectroscopy (^1^H-MRS) provides a noninvasive method of assessing an *in vivo* biochemistry of brain tissue. This review summarizes the main results obtained from the application of neuroimaging techniques in DLB cases focusing on ^1^H-MRS.

## 1. Introduction

Dementia with Lewy bodies (DLB) is considered to be the second most frequent primary degenerative dementing illness after Alzheimer's disease (AD). According to some investigators from research centres and brain banks it comprises up to 20% of all dementia cases [1]. DLB, together with Parkinson's disease (PD), Parkinson's disease with dementia (PDD) belong to *α*-synucleinopathies—a group of neurodegenerative diseases related by the pathological accumulation of the *α*-synuclein protein [2, 3]. The clinical and neuropathologic criteria for DLB and consensus criteria were published by McKeith et al. [4–7].

DLB is characterized by dementia (a progressive cognitive decline with deficits in attention, executive functions, and visuospatial ability associated with fluctuations), visual hallucinations, and parkinsonism. Presence of parkinsonism, visual hallucinations, and specific profile of cognitive deficits allows for differential diagnosis with AD or frontotemporal lobar degeneration (FTLD), especially at the early stage of disease. Dementia due to PD and DLB shares clinical symptoms and neuropsychological profiles. Moreover, the core features and additional clinical signs and symptoms for these two very similar diseases are largely the same. Researchers have spent over decade debating whether these are two different diseases or simply different phenotypes of one single entity. PDD and DLB are separated mostly by the “one-year rule” of dementia onset, which is frequently the only criterion applied in differential diagnosis. It seems that the temporal sequence of symptoms and clinical features of PDD and DLB justify distinguishing these disorders. Details on doubts and boundary issues are well covered in the paper by Lippa et al. [8] and review on clinical presentation of DLB was published lately by Morra and Donovick [9].

Conflicting data are present on cognitive decline rate and duration of the illness. In the study by Williams et al. [10] DLB was characterised by increased risk of death compared with AD, but the two groups did not differ in rate of cognitive decline. More rapid progression of cognitive decline and shorter duration of dementia were found in DLB in comparison to AD in the naturalistic study by Magierski et al. [11], but no differences between these two types of dementia in the rate of progression were found in other studies [12].

Accurate* ante mortem* diagnosis of DLB is essential for several reasons. First, the detailed and exact diagnosis of dementia subtype is needed in clinical studies on efficacy and safety of treatment. Second, current treatment options that are effective in one type of dementia may not be useful or dangerous in other types [13, 14]. A patient with DLB usually responds well to cholinesterase inhibitors [15–17] and improvement in some neuropsychiatric symptoms was confirmed [18]. The antipsychotic treatment is known to be a dangerous treatment option in DLB because of the risk of exacerbation of extrapyramidal symptoms and is generally contraindicated in this disorder [19–21], but in the study by Johnell at al. [22] the use of antipsychotics in DLB patients was surprisingly high (16% in DLB patients) with an adjusted odds ratio of 4.2 compared to AD patients. Third, in clinical studies it is essential to identify uniform diagnostic groups. According to Watson et al. [23] in light of the poor sensitivity of the consensus criteria, it is important to establish additional markers which, when combined with clinical assessment, can improve diagnostic accuracy. Vernon et al. [24] stated that there is no clear diagnostic imaging marker that offers a reliable differential diagnosis between the different forms of Lewy body diseases (PD, PDD, or DLB) or that could facilitate tracking of disease progression.

Neuroimaging seems to be an obvious method which allows obtaining additional information on brain structure, changes, and functioning. The development of imaging methods or other objective measures to supplement clinical criteria is needed. However, a method which would accurately facilitate diagnosis of DLB prior to death is still being searched.

## 2. Imaging in Dementia with Lewy Bodies

Neuroimaging seems to be a promising method in dementia studies. Both structural imaging research and functional imaging research have been performed in DLB patients [25]. Most imaging studies on DLB have used standard structural magnetic resonance imaging (MRI). Also, other MRI techniques, such as tensor-diffusion imaging to visualize fiber tracts, MRI spectroscopy to visualize* in vivo* metabolism, and magnetization transfer ratios to visualize fine structural damage, have been studied in DLB patients [26].

The main structural imaging finding assumed as characteristic change in the DLB cases is the relative preservation of hippocampal and medial temporal lobe, a feature that is important in its differentiation from AD. White matter lesions are equally frequent in DLB and AD and together with cortical pathology may influence the severity of cognitive impairment.

The detailed description of findings in neuroimaging studies in DLB and the role of different MRI based techniques is covered in the excellent paper by Watson et al. [23].

Functional examination of the brain tissue is available with 18F-fluorodeoxyglucose positron emission tomography (FDG-PET) or single photon emission computed tomography (SPECT) and both have clinical utility for the differential diagnosis of dementia [27]. Occipital hypometabolism in PET and hypoperfusion in SPECT were observed in DLB, while temporal lobe perfusion is relatively preserved [28]. Intense research was done for visualizing brain neurotransmitter's abnormalities in DLB, especially in the dopaminergic system [29] and reduced dopamine transporter levels in DLB as shown with [123I]FP-CIT-SPECT currently appear to be the most reliable and valid biomarker of disease [30]. ^123^I-MIBG myocardial scintigraphy may have an important position in differential diagnosis between DLB and other dementias and for that purpose it was included into the diagnostic criteria of DLB [7].

Neurochemical studies have shown a pronounced reduction in the cholinergic activity in DLB, even greater than in AD brains. Investigation of brain metabolites changes is challenging in the context of neuropathological and neurochemical findings [31].

## 3. Proton Magnetic Resonance Spectroscopy (^**1**^H-MRS)

Proton magnetic resonance spectroscopy (^1^H-MRS) provides a noninvasive method of assessing an* in vivo* biochemistry of brain tissue. ^1^H-MRS using standard or research-dedicated magnetic resonance imaging devices allows making measurements of chemical levels within the brain by measuring the signal originating from protons attached to key biomolecules. The neurochemistry is defined on a regional basis by acquiring a radiofrequency signal with chemical shift from one or many volumes or voxels. The result of ^1^H-MRS examination is a spectrum and up to 80 brain metabolites and flux rates can be distinguished within the spectrum [36]. The signal indicating particular compound is localized on a horizontal scale (chemical shift), and their relative metabolite concentration is determined from the metabolite's peak height. The brain proton spectrum includes metabolite peaks for 5 important compounds: N-acetylaspartate (NAA), creatine (Cr), choline (Cho), myo-inositol (mI), and glutamine/glutamate (Glx). Peaks of lipids and lactate are not observed in healthy brain, and therefore their absorptions are not visible within normal spectrum. Both have diagnostic value in cases of brain diseases. NAA is regarded as a marker of neuronal integrity and is reduced in neuronal dysfunction or loss. Creatine is a marker of general metabolism and is assumed to be relatively constant. Therefore, a peak of Cr is often applied as an internal reference level and is used for ratio's calculation. Choline is a metabolic marker of membrane density and integrity. Myo-inositol is mainly present in the glial cells and is considered as a glial marker. Finally, glutamine/glutamate metabolism occurs in neurons and glial cells and plays a role in detoxification and regulation of neurotransmitters. Reduction of glutamine/glutamate (Glx) may reflect glial cell or axonal impairment. The detailed description of MRS technique basics is covered excellently elsewhere [37–41].

Among all dementia types, ^1^H-MRS was firstly used in the studies on AD and MCI. Both decreased N-acetylaspartate (NAA) and increased myo-inositol in the occipital, temporal, parietal, and frontal regions as well as in whole brain of AD patients were found [42, 43] and changes were detectable even at the early stages of the disease. ^1^H-MRS examination was used for identifying MCI, distinguishing between MCI and normal controls [44, 45] and result of examination was evaluated as a predictor of clinical conversion of MCI to AD dementia based on clinical followup [46, 47]. The most current paper on MRS in MCI, summarizing 29 papers and providing meta-analysis of data, was published by Tumati et al. last year [48].

Proton magnetic resonance spectroscopy was used for determination profile of brain metabolites in DLB, but limited published data of ^1^H-MRS are available in DLB patients in comparison to AD or MCI. Only 4 original papers including DLB cases were identified and all of them are described later (see Table 1). Cause of this lacking research in DLB cases is complex and not fully understood. Among all, duration of examination with 1.5T MRI scanner seems difficult and not feasible in many DLB patients. Brain atrophy, cognitive fluctuation, psychotic symptoms, and motor artefacts due to Parkinsonian features in particular are the reasons for difficulties in ^1^H-MRS studies in DLB subjects.

The first ^1^H-MRS study in DLB subjects was published in 2002. Molina et al. [32] examined white matter from the left centrum semiovale and grey matter from the midline parietal region in DLB patients and age-matched healthy controls. Investigators made an attempt to acquire spectra from the temporal lobe and basal ganglia. These measurements were unsuccessful due to lack of proper magnetic homogeneity in those regions in almost all patients and many of the healthy controls. Authors reported significantly lower mean NAA/Cr, Glx1/Cr, and Cho/Cr ratios in the white matter. No significant differences in the grey matter were found. Finally, authors concluded that the large overlap between the spectroscopic profiles of DLB patients and healthy subjects limits usefulness of this method in the differentiation procedure.

We have performed pilot study to evaluate the feasibility of proton magnetic resonance spectroscopy in DLB and so far results were published in part as a conference poster only [34]. Primarily, 22 subjects meeting the Consortium on DLB International Workshop Criteria for probable DLB were evaluated. DLB patients represented different dementia stages, so the DLB group was not homogeneous. Finally, 15 DLB subjects and 14 patients meeting DSM-IV criteria for AD were included and final results are described below. Seven DLB subjects were excluded because they were severely demented and uncooperative, even during clinical assessment. Eleven healthy control subjects participated in the study.

The subjects included in the study underwent general medical, neurological, psychiatric, and neuropsychological investigations. The clinical assessment included vital signs, the mini-mental state examination, clinical dementia rating (CDR), the clock drawing test, Hachinski ischemic scale, the motor section of the unified Parkinson's disease rating scale (UPDRS), the memory-orientation-concentration test of Blessed, the neuropsychiatric inventory (NPI), and the activities of daily living (ADL). The neuropsychological assessment consisted of the evaluation of short-term memory (forward and backward digit span), episodic memory (10-items word list), semantic memory (Boston naming test, letter, and category fluency tests), perceptual and spatial abilities (WAIS-R block designs subtest, Rey complex figure), and abstractive reasoning (WAIS-R similarities subtest). Diagnostic criteria were applied and AD and DLB groups were selected on the basis of clinical and neuropsychological assessment. Control subjects were recruited in the group of patients' relatives. Volunteers underwent psychiatric and neuropsychological investigations. None of the participating control subjects had any neurological or somatic diseases. All demented patients in this study had an informant who provided an adequate clinical history.

All patients and volunteers were examined using a 1.5 T MR scanner with a head coil. We performed MRI in T1 weighted images, in three orthogonal planes without administration of paramagnetic contrast medium. These images were used for voxel positioning. ^1^H-MRS was performed using short echo time SVS STEAM sequence: TE 20 ms and TR 2000 ms. Volumes of interest (VOI, voxel) were positioned in the parietal white matter, occipital grey matter, and temporal lobe separately. The raw data were then evaluated automatically with the protocols available in the Magnetom Vision Plus—Siemens software. The relative signal intensities of the main metabolites were obtained by manual and semiautomated approximations of the spectra chosen from the volumes of interest. The ratios of the metabolites relative signal intensities in the group of healthy volunteers were evaluated separately for the brain parietal white matter, occipital grey matter and temporal lobe. These ratios were used as a reference to determine the metabolite changes occurring in patients with DLB and AD.

In our study, ^1^H-MRS scans acquiring in 3 localizations was successful in 5 DLB, 7 AD patients and 7 control subjects. Five DLB subjects were uncooperative during scanning so measurement was ineffective in all localizations. Examination of the temporal lobe failed because of movement artefacts in 2 AD patients. ^1^H-MRS scans acquiring was successful in the centrum semiovale in all AD and control subjects and 9 DLB patients. Measurement of metabolites in the occipital lobe was successful in all AD and control subjects and 10 DLB patients. Attempts were made to acquire proton spectra from the temporal lobes. These measurements were unsuccessful due to voxel localization problems associated with large brain atrophy in this region in 5 AD and 4 DLB patients and 4 volunteers. Examination of the occipital lobe and white matter failed in the more impaired subjects. This group had higher NPI (OL: *P* = 0.013 and WM: *P* = 0.044, resp.) and UPDRS (OL: *P* = 0.001 and WM: *P* = 0.003, resp.) than the group with successful examination.

In our study, temporal lobe scanning was unsuccessful in 21 cases; the reasons for difficulties were uncooperativeness in 5 DLB cases, movement artefacts in 2 AD and 1 DLB subjects, and pronounced brain atrophy in the examined region in 5 AD and 4 DLB patients and 4 controls. Movement artefacts were caused by various factors, primarily by Parkinsonism of DLB subjects. Features of Parkinsonism are generally mild to moderate in DLB but usually start unanimously with dementia. Bradykinesia, rigidity and falls are common, while resting tremor could be absent. Rigidity makes it very difficult to lie on the back for a long time. The structure of the examination procedure is probably the second cause of movement artefacts. The temporal lobe was examined following the examination of centrum semiovale and occipital region, which might have caused patients fatigue and inability to remain motionless. The complete procedure is 40 minute long and could be exhausting for the demented subjects. Moreover, uncooperativeness of DLB subjects could be a consequence of fluctuations of cognition.

Unsuccessful scanning of the temporal lobe in our study was caused by considerable brain atrophy in the examined region in 5 AD and 4 DLB patients and 4 volunteers. Different patterns of brain atrophy were described by Burton et al. [49]. They observed regional grey matter loss bilaterally in the temporal and frontal lobes and insular cortex of DLB patients compared to control subjects. Comparison of dementia groups showed preservation of the medial temporal lobe, hippocampus, and amygdala in DLB relative to AD.

We have not found any correlation between unsuccessful temporal lobe scanning and any analysed variable (cognitive impairment, NPI score, and UPDRS score). Excessive asymptomatic brain atrophy could account for this finding. It was also seen in the control group, where we have found the atrophy of the temporal lobe without clinical impairment during neuropsychological assessment. Parkinsonism and behavioural disturbances made scanning of the centrum semiovale and occipital lobe difficult, without negative effect on the temporal lobe examination. The assessment of centrum semiovale and occipital lobe in DLB patients was more difficult than in AD and control groups.

Kantarci et al. [33] evaluated ^1^H-MRS metabolite ratio changes in common dementias (AD, DLB, FTLD, and vascular dementia (VaD)) with respect to normal subjects within standard voxels covering right and left posterior cingulate gyri and inferior precunei. The study showed a number of differences in the ^1^H-MRS metabolites profiles and it will be discussed in detail below. NAA/Cr ratio lower than normal in patients with AD, FTLD, and VaD was reported. They found lower NAA/Cr ratio in AD and FTLD cases than in DLB patients. mI/Cr ratio was higher in patients with AD and FTLD than in normal subjects. In patients with AD, FTLD, and DLB higher Ch/Cr ratio was found when compared to normal subjects. No metabolite differences between patients with AD and FTLD or between patients with DLB and VaD were found. mI/Cr ratio was higher in patients with AD and FTLD than VaD. Moreover, mI/Cr was higher in patients with FTLD than DLB too. The only measurement that was different from normal in patients with DLB was the Cho/Cr ratio. Authors concluded that they found decreased level of NAA/Cr in dementias characterized by neuron loss (AD, FTLD, and VaD). As it could be expected, mI/Cr levels were increased in dementias associated with gliosis (AD and FTLD). Finally, Cho/Cr levels were elevated in dementias with a profound cholinergic deficit (AD and DLB). In discussion the authors stated that the elevation of Cho/Cr in AD and DLB patients is not completely understood and requires further research. It can be linked to decrease of Cho/Cr levels with cholinergic agonist treatment in AD, so they suggested that Cho/Cr levels could be a biomarker of therapeutic efficacy in AD and DLB drug trials.

Xuan et al. [35] assumed that the decrease of NAA in hippocampus was found in studies of AD patients, so the same result may be found in DLB patients. DLB patients showed statistically significant reduction in NAA/Cr ratios in left hippocampus when compared to controls. Cho/Cr ratios of DLB subjects did not differ from those of the control group. NAA/Cr ratios of DLB patients in right hippocampus were also significantly lower than controls. Similarly to the left side, Cho/Cr ratios in right hippocampus of DLB patients did not differ from those of the control group. Authors concluded that their data show relatively decrease of N-acetylaspartate in the hippocampus of patients with early- or intermediate-stage DLB.

Another interesting aspect of ^1^H-MRS examination is prognostic value of changes in brain metabolic concentrations. Attempts for selecting MCI cases who will convert to DLB or AD were made. Fayed et al. [50] described group of MCI cases (*n* = 119) who were examined with ^1^H-MRS at the baseline visit and were monitored through followup. After the followup period (a mean period of 29 months; range 17–44), 54 patients converted to dementia (AD, *n* = 49; DLB, *n* = 5). Metabolites ratios were compared, but they did not find differences in NAA/Cr ratio or Cho/Cr ratios between patients with DLB and patients with other types of MCI or AD. In contrast, Zhang et al. [51] reported that decreased NAA/Cr ratio in the posterior cingulate gyri characterized patients with MCI who progressed to AD and distinguished them from MCI patients who progressed to DLB. So they concluded that ^1^H-MRS may be a useful adjunct in early differential diagnosis of AD and DLB in patients with MCI.

Some data were published in the field of proton spectroscopy in Parkinson's disease dementia. As it was stated above, PDD and DLB share many clinical and neuropsychological features. So it could be challenging to compare results of DLB and PDD studies made with ^1^H-MRS technique. Maybe it will be possible to confirm or reject assumption that clinicians are able to separate PDD and DLB mostly by the “one-year rule” of dementia onset. In other words, maybe the ^1^H-MRS spectrum is a candidate for winning the title of noninvasive and precise biomarker.

So far, significantly decreased level of NAA in the occipital region in the PDD group compared to the PD and control groups was found [52]. Significantly, lower NAA/Cr ratio of the posterior cingulate in PDD cases when compared with controls and nondemented PD patients was also found in study by Griffith et al. [53]. Authors observed no abnormalities in Cho/Cr or mI/Cr ratios of PDD cases [53], but changes were visible when comparison of AD and controls was done [54]. Significantly reduced NAA/Cr ratio and significantly increased Cho/Cr ratio and mI/Cr ratio of posterior cingulate in AD cases when compared to controls were described. Moreover, patients with PDD exhibited significantly reduced NAA/Cr and Glu/Cr ratios compared with controls. Glu/Cr ratio was also significantly reduced in PDD cases compared with AD. The findings suggest that reduced NAA/Cr of the posterior cingulate could be used as a marker for dementia in patients with PD, authors said in conclusion [53].

Interestingly, changes in metabolites correlated with different aspects of clinical status of PDD and PD cases. The correlation between NAA/Cr ratio and mental status of patients with PD and patients with PDD was observed [53] and NAA values correlated with neuropsychological performance but not with severity of motor impairment [52]. Lately, similar metabolic and clinical findings were described by Pagonabarraga et al. [55] who examined spectrum of PD patients (cognitively intact cases, patients with mild cognitive impairment, and cases with dementia). They have analyzed the relative importance of temporal lobe defects versus executive impairment in the progression to dementia in PD by using ^1^H-MRS of the hippocampus and dorsolateral prefrontal cortex. NAA concentrations in the right dorsolateral prefrontal cortex were significantly decreased in PD cases with MCI when compared to cognitively intact PD cases. NAA concentrations were also significantly decreased in the left hippocampus of PDD cases when compared to PD patients with MCI. Similarly to previous studies, decrease of NAA was correlated with neuropsychological results.

## 4. Conclusions

Many authors focused their research or papers on differential diagnosis in dementia. As it was said in the introduction exact diagnosis is essential for many purposes. At this moment there are so many similarities and only some differences between DLB and PDD, both Lewy body diseases. Attempts were done for establishing biomarkers with CSF examination [56, 57], neuroimaging, and neurochemistry [58].

Proton magnetic resonance spectroscopy (^1^H-MRS) provides a noninvasive method of assessing an* in vivo* tissue biochemistry. ^1^H-MRS using standard or research-dedicated magnetic resonance imaging devices does not need any injection of contrast substances, and the price of evaluation is comparable with regular MRI examination (few times lower than PET scanning). ^1^H-MRS seems to be a promising method of brain research and was intensively applied in many neurological disorders, including AD cases. Studies in DLB and PDD were also performed but are limited in number. To our knowledge, papers with head-to-head comparison of DLB and PDD cases are lacking and there is a need for further studies. Honestly speaking this could be challenging due to difficulties described earlier.

Last but not least, differential diagnosis is essential at the beginning of treatment and at early clinical presentation. At early phase of dementing illness it is easier, and in some cases it is possible at this time only to make differential diagnosis based on clinical and neuropsychological evaluation. Within the course of dementia major symptoms disappear, additional symptoms occur (for example, seizures or Parkinsonism), and AD may mimic Lewy body diseases. Neuropathological verification of clinical diagnosis has the best value. It would be perfect for differential diagnosis and for understanding of the nature of DLB to perform ^1^H-MRS examination at MCI stage, follow the cases to the point of dementia diagnosis, and verify diagnosis postmortem.

## Figures and Tables

**Table 1:** 1H-MRS studies in DLB (modified after Watson et al. [23]).

Author and reference	Subjects	Area (voxel of interest)	Metabolite ratios in DLB cases			
			NAA/Cr	Cho/Cr	Glx/Cr	mI/Cr
Molina et al. (2002) [32]	CTL (*n* = 11) vs DLB (*n* = 12)	WM: centrum semiovale GM: parasagittal parietal cortex	↓ *↔*	↓ *↔*	↓ *↔* *↔*	NA NA
Kantarci et al. (2004) [33]	CTL (*n* = 206) vs DLB (*n* = 20) vs AD (*n* = 121)	Right and left posterior cingulated gyrus and inferior precunei	*↔*	↑ *↔*	*↔*	*↔*
Magierski et al. (2004) [34]	CTL (*n* = 8) vs DLB (*n* = 12) vs AD (*n* = 12)	Centrum semiovale, occipital grey matter and temporal lobes	↓	NA	NA	*↔*
Xuan et al. (2008) [35]	CTL (*n* = 8) vs DLB (*n* = 8)	Bilateral hippocampi	↓	*↔*	NA	NA

*↔*: Unchanged; ↓: reduced; ↑: increased; NA: not applicable; CTL: controls; AD: Alzheimer's disease; DLB: dementia with Lewy bodies; WM: white matter; GM; grey matter.

## Abbreviations

DLB: Dementia with Lewy Bodies
AD: Alzheimer's disease
PDD: Parkinson's disease dementia
MCI: Mild cognitive impairment
CTL: Controls
FTLD: Frontotemporal lobar degeneration
VaD: Vascular dementia
^1^H-MRS: Proton magnetic resonance spectroscopy
MRI: Magnetic resonance imaging
PET: Positron emission tomography
SPECT: Single photon emission computed tomography
NAA: N-acetylaspartate
Cho: Choline compound
Cr: Creatine and phosphocreatine
mI: Myo-inositol
Glx: Glutamate-glutamine complex
Ppm: Parts per million.
